# Supplementary material for: DR4/DQ2 haplotype confers susceptibility to T1DM with early clinical disease onset: A retrospective analysis in a tertiary-care hospital in Italy
Source: PLoS One. 2022 Nov 21;17(11):e0276896. doi: 10.1371/journal.pone.0276896 (PMC9678300; doi:10.1371/journal.pone.0276896)
Supplement: S1 Table — (DOCX) [file pone.0276896.s001.docx]

**S1 Table.** All demographic, clinical and serological details for 163 subjects affected by T1DM with HLA high-resolution

| **Subject number** | **Sex** | **Haplotype** | **Origin** | **Age at T1DM onset (years)** | **Autoimmune comorbidity and selective IgA deficiency** | **T1DM familiarity** | **Autoantibodies**  **(IA2 or IAA or anti-GAD)** |
| --- | --- | --- | --- | --- | --- | --- | --- |
| 1 | f | DRB1*0402, DQB1*0302; DQA1*03 | Not known | 17,1 | Autoimmune thyroiditis |  | + |
| 2 | m | DRB1*03, DQB1*0201, DQA1*0501 | Not known | 3,9 |  |  | + |
| 3 | m | DRB1*03, DQB1*0201, DQA1*0501; DRB1*0402, DQB1*0302, DQA1*03 | Not known | 2,9 |  |  | + |
| 4 | m | DRB1*03, DQB1*0201, DQA1*0505 DRB1*0405, DQB1*0302, DQA1*03 | Italy | 12,4 |  |  | + |
| 5 | f | DRB1*03, DQB1*0201, DQA1*0501 DRB1*0402, DQB1*0302, DQA1*03 | Italy | 7,1 | Celiac disease, autoimmune thyroiditis, selective IgA deficiency |  | + |
| 6 | m | DRB1*03, DQB1*0201, DQA1*0501 DRB1*0404, DQB1*0302, DQA1*03 | Italy | 7,0 |  |  | + |
| 7 | m | DRB1*03, DQB1*0201, DQA1*0501 | Italy | 12,3 |  |  | + |
| 8 | m | DRB1*03, DQB1*0201, DQA1*0501 | Italy | 7,8 |  |  | + |
| 9 | m | DRB1*03, DQB1*0201, DQA1*0501 | Italy | 10,0 | Celiac disease |  | + |
| 10 | f | DRB1*03, DQB1*0201, DQA1*0501 DRB1*0405, DQB1*0302, DQA1*03 | Italy | 1,0 | Celiac disease |  | + |
| 11 | f | DRB1*03, DQB1*0201, DQA1*0501 | Italy | 12,1 |  |  | + |
| 12 | f | DRB1*03, DQB1*0201, DQA1*0505 DRB1*0402, DQB1*0302, DQA1*03 | Italy | 14,1 | Autoimmune thyroiditis |  | + |
| 13 | f | DRB1*03, DQB1*0201, DQA1*0501 DRB1*0404, DQB1*0302, DQA1*03 | Italy | 12,3 |  |  | + |
| 14 | m | DRB1*03, DQB1*0201, DQA1*0501 DRB1*0402, DQB1*0302, DQA1*03 | Italy | 3,1 |  |  | + |
| 15 | f | DRB1*03, DQB1*0201, DQA1*0501 | Italy | 6,6 |  | T1DM familiarity (father) | + |
| 16 | f | DRB1*0401, DQB1*0302, DQA1*03/X | Italy | 10,2 |  |  | + |
| 17 | m | DRB1*0401, DQB1*0302, DQA1*03/X | Romania | 5,1 |  |  | + |
| 18 | m | DRB1*03, DQB1*0201, DQA1*0501 DRB1*0401, DQB1*0302, DQA1*03 | Italy | 14,2 |  |  | + |
| 19 | m | DRB1*03, DQB1*0201, DQA1*0501 | Italy | 5,6 |  |  | + |
| 20 | m | DRB1*03, DQB1*0201, DQA1*0501 DRB1*0402, DQB1*0302, DQA1*03 | Italy | 9,9 |  |  | + |
| 21 | m | DRB1*03, DQB1*0201, DQA1*0501 | Italy | 16,7 | Autoimmune thyroiditis |  | + |
| 22 | m | DRB1*03, DQB1*0201, DQA1*0501 | China | 7,6 |  |  | + |
| 23 | m | DRB1*03, DQB1*0201, DQA1*0501 | Italy | 1,2 | Celiac disease; |  | + |
| 24 | f | DRB1*03, DQB1*0201, DQA1*0501 | Italy | 8,4 |  |  | + |
| 25 | m | DRB1*03, DQB1*0201, DQA1*0501 | Italy | 15,0 | Selective IgA deficiency |  | + |
| 26 | m | DRB1*0404, DQB1*0302, DQA1*03/X | Italy | 11,8 |  |  | + |
| 27 | m | DRB1*03, DQB1*0201, DQA1*0501 | Italy | 11,9 |  |  | + |
| 28 | m | DRB1*03, DQB1*0201, DQA1*0501 DRB1*0401, DQB1*0302, DQA1*03 | Italy | 7,1 | Celiac disease |  | + |
| 29 | m | DRB1*03, DQB1*0201, DQA1*0501 DRB1*0404, DQB1*0302, DQA1*03 | Italy | 5,0 |  |  | + |
| 30 | m | DRB1*03, DQB1*0201, DQA1*0501 DRB1*0404, DQB1*0302, DQA1*03 | Italy | 14,1 |  |  | + |
| 31 | f | DRB1*03, DQB1*0201, DQA1*0501 | Italy | 11,9 |  | T1DM familiarity (father) | + |
| 32 | m | DRB1*0401, DQB1*0302, DQA1*03/X | Italy | 13,0 |  |  | + |
| 33 | f | DRB1*03, DQB1*0201, DQA1*0501 | Albania | 8,0 |  |  | + |
| 34 | m | DRB1*03, DQB1*0201, DQA1*0501 DRB1*0405, DQB1*0302, DQA1*03 | Italy | 10,2 | Autoimmune thyroiditis |  | + |
| 35 | m | DRB1*0404, DQB1*0302, DQA1*03/X | Italy | 14,1 |  |  | + |
| 36 | f | DRB1*03, DQB1*0201, DQA1*0501 | Italy | 2,6 | Autoimmune thyroiditis |  | + |
| 37 | m | DRB1*03, DQB1*0201, DQA1*0501 | Italy | 9,0 |  |  | + |
| 38 | m | DRB1*03, DQB1*0201, DQA1*0501 DRB1*0404, DQB1*0302, DQA1*03 | Italy | 12,8 | Celiac disease |  | + |
| 39 | m | DRB1*03, DQB1*0201, DQA1*0501 DRB1*0405, DQB1*02, DQA1*03 | Albania | 6,0 |  |  | + |
| 40 | m | DRB1*03, DQB1*0201, DQA1*0501 DRB1*0405, DQB1*0302, DQA1*03 | Afghanistan | 11,3 |  |  | + |
| 41 | m | DRB1*03, DQB1*0201, DQA1*0501 DRB1*0405, DQB1*0302, DQA1*03 | Italy | 4,1 | Celiac disease |  | + |
| 42 | f | DRB1*0402, DQB1*0302, DQA1*03 DRB1*0402, DQB1*0302, DQA1*03 | Italy | 10,9 |  |  | + |
| 43 | f | DRB1*03, DQB1*0201, DQA1*0501 | Italy | 15,7 | Selective IgA deficiency – Celiac disease |  | + |
| 44 | f | DRB1*03, DQB1*0201, DQA1*0501 | Italy | 4,2 | Celiac disease |  | + |
| 45 | f | DRB1*03, DQB1*0201, DQA1*0501 DRB1*0404, DQB1*0302, DQA1*03 | Italy | 12,6 |  |  | + |
| 46 | m | DRB1*03, DQB1*0201, DQA1*0501 | Morocco | 3,1 |  |  | + |
| 47 | f | DRB1*03, DQB1*0201, DQA1*0501 | Not known | 7,7 |  |  | + |
| 48 | f | DRB1*03, DQB1*0201, DQA1*0501 | Albania | 15,3 | Autoimmune thyroiditis |  | + |
| 49 | m | DRB1*03, DQB1*0201, DQA1*0501 | Italy | 10,3 |  |  | + |
| 50 | m | DRB1*03, DQB1*0201, DQA1*0501 | Italy | 13,0 | Autoimmune thyroiditis | T1DM familiarity (father) | + |
| 51 | m | DRB1*03, DQB1*0201, DQA1*0501 | Italy | 5,9 | Celiac disease |  | + |
| 52 | m | DRB1*03, DQB1*0201, DQA1*0501 | Not known | 4,1 |  |  | + |
| 53 | f | DRB1*03, DQB1*0201, DQA1*0501 DRB1*0405, DQB1*0302, DQA1*03 | Morocco | 9,5 |  |  | + |
| 54 | m | DRB1*0402, DQB1*0302, DQA1*03/X | Morocco | 4,5 |  |  | + |
| 55 | f | DRB1*03, DQB1*0201, DQA1*0501 | Italy | 9,2 | Celiac disease |  | + |
| 56 | f | DRB1*03, DQB1*0201, DQA1*0501 DRB1*0405, DQB1*0302, DQA1*03 | Italy | 1,5 | Celiac disease | T1DM familiarity (father) | - |
| 57 | f | DRB1*03, DQB1*0201, DQA1*0501 DRB1*0401, DQB1*0302, DQA1*03 | Italy | 6,9 | Celiac disease |  | + |
| 58 | f | DRB1*03, DQB1*0201, DQA1*0501 | Italy | 10,1 | Autoimmune thyroiditis |  | + |
| 59 | m | DRB1*03, DQB1*0201, DQA1*0501 | Italy | 12,5 |  |  | + |
| 60 | f | DRB1*0404, DQB1*0302, DQA1*03/X | Italy | 14,1 |  |  | + |
| 61 | f | DRB1*03, DQB1*0201, DQA1*0501 | Italy | 4,4 | Celiac disease |  | + |
| 62 | f | DRB1*03, DQB1*0201, DQA1*0501 | Italy | 10,8 |  |  | + |
| 63 | f | DRB1*03, DQB1*0201, DQA1*0501 DRB1*0401, DQB1*0302, DQA1*03 | Italy | 12,2 |  |  | + |
| 64 | f | DRB1*0402, DQB1*0302, DQA1*03/X | Italy | 9,2 |  |  | + |
| 65 | m | DRB1*03, DQB1*0201, DQA1*0501 DRB1*0401, DQB1*0302, DQA1*03 | Italy | 5,2 |  |  | + |
| 66 | f | DRB1*0401, DQB1*0302, DQA1*03/X | Italy | 17,5 | Autoimmune thyroiditis |  | + |
| 67 | f | DRB1*0405, DQB1*0302, DQA1*03/X | Italy | 16,5 |  |  | + |
| 68 | f | DRB1*03, DQB1*0201, DQA1*0501 | Italy | 11,4 |  |  | + |
| 69 | f | DRB1*03, DQB1*0201, DQA1*0501 | Italy | 10,0 | Celiac disease |  | + |
| 70 | f | DRB1*03, DQB1*0201, DQA1*0505 DRB1*0405, DQB1*0302, DQA1*03 | Italy | 9,6 |  |  | + |
| 71 | m | DRB1*03, DQB1*0201, DQA1*0501 DRB1*0402, DQB1*0302, DQA1*03 | Italy | 1,7 |  |  | + |
| 72 | f | DRB1*03, DQB1*0201, DQA1*0501 | Italy | 10,0 |  |  | + |
| 73 | m | DRB1*03, DQB1*0201, DQA1*0501 | Italy | 7,2 |  |  | + |
| 74 | m | DRB1*03, DQB1*0201, DQA1*0501 | Italy | 6,3 | Autoimmune thyroiditis |  | + |
| 75 | m | DRB1*03, DQB1*0201, DQA1*0501 | Italy | 4,9 |  |  | + |
| 76 | f | DRB1*0405, DQB1*0302, DQA1*03/X | Italy | 10,8 |  |  | + |
| 77 | f | DRB1*03, DQB1*0201, DQA1*0501 | Italy | 6,2 |  |  | + |
| 78 | f | DRB1*03, DQB1*0201, DQA1*0501 | Italy | 13,1 |  |  | + |
| 79 | m | DRB1*03, DQB1*0201, DQA1*0501 DRB1*0401, DQB1*0302, DQA1*03 | Italy | 12,2 |  |  | + |
| 80 | m | DRB1*03, DQB1*0201, DQA1*0501 | Italy | 8,4 |  |  | + |
| 81 | f | DRB1*0402, DQB1*0302, DQA1*03/X | Italy | 11,4 |  |  | + |
| 82 | m | DRB1*03, DQB1*0201, DQA1*0501 | Italy | 8,4 |  |  | + |
| 83 | m | DRB1*03, DQB1*0201, DQA1*0501 | Italy | 15,2 |  |  | - |
| 84 | m | DRB1*03, DQB1*0201, DQA1*0501 | Italy | 10,5 |  | T1DM familiarity (sister or brother) | - |
| 85 | m | DRB1*03, DQB1*0201, DQA1*0501 | Italy | 10,4 |  | T1DM familiarity (sister or brother) | - |
| 86 | f | DRB1*0401, DQB1*0302, DQA1*03/X | Italy | 10,4 | Autoimmune thyroiditis |  | + |
| 87 | m | DRB1*03, DQB1*0201, DQA1*0501 | Italy | 5,2 | Celiac disease |  | + |
| 88 | f | DRB1*03, DQB1*0201, DQA1*0505 DRB1*0402, DQB1*0302, DQA1*03 | Italy | 13,0 |  |  | + |
| 89 | f | DRB1*0402, DQB1*0302, DQA1*03/X | Italy | 12,6 |  |  | + |
| 90 | m | DRB1*03, DQB1*0201, DQA1*0501 DRB1*0405, DQB1*0302, DQA1*03 | Italy | 5,1 |  |  | + |
| 91 | m | DRB1*0404, DQB1*0302, DQA1*03/X | Italy | 11,7 |  |  | + |
| 92 | m | DRB1*0405, DQB1*0302, DQA1*03/X | Italy | 6,2 |  |  | + |
| 93 | f | DRB1*03, DQB1*0201, DQA1*0501 DRB1*0401, DQB1*0302, DQA1*03 | Italy | 11,5 | Autoimmune thyroiditis |  | + |
| 94 | m | DRB1*0401, DQB1*0302, DQA1*03/X | Italy | 12,3 |  |  | + |
| 95 | f | DRB1*03, DQB1*0201, DQA1*0501 | Italy | 12,0 |  |  | + |
| 96 | f | DRB1*0401, DQB1*0302, DQA1*03/X | Italy | 8,8, |  |  | + |
| 97 | m | DRB1*03, DQB1*0201, DQA1*0501 | Italy | 15,4 |  |  | + |
| 98 | m | DRB1*03, DQB1*0201, DQA1*0505 DRB1*0405, DQB1*0302, DQA1*03 | Not known | 5,8 |  |  | + |
| 99 | m | DRB1*03, DQB1*0201, DQA1*0501 | India | 2,7 |  | T1DM familiarity (father) | + |
| 100 | f | DRB1*03, DQB1*0201, DQA1*0501 DRB1*0404, DQB1*0302, DQA1*03 | India | 6,3 | Celiac disease |  | + |
| 101 | m | DRB1*03, DQB1*0201, DQA1*0501 | India | 8,6 |  |  | + |
| 102 | f | DRB1*03, DQB1*0201, DQA1*0501 DRB1*0405, DQB1*0302, DQA1*03 | Italy | 13,7 |  |  | + |
| 103 | m | DRB1*0402, DQB1*0302, DQA1*03 DRB1*0405, DQB1*0302, DQA1*03 | Italy | 13,2 | Celiac disease |  | + |
| 104 | m | DRB1*03, DQB1*0201, DQA1*0501 DRB1*0401, DQB1*0302, DQA1*03 | Italy | 13,9 |  |  | + |
| 105 | f | DRB1*0405, DQB1*0302, DQA1*03/X | Italy | 13,3 | Celiac disease; autoimmune thyroiditis | T1DM familiarity (sister or brother) | + |
| 106 | f | DRB1*0405, DQB1*0302, DQA1*03/X | Italy | 13,8 | Autoimmune thyroiditis | T1DM familiarity (sister or brother) | + |
| 107 | m | DRB1*03, DQB1*0201, DQA1*0501 | Italy | 8,5 | Celiac disease |  | + |
| 108 | m | DRB1*03, DQB1*0201, DQA1*0501 | Poland | 6,8 |  |  | + |
| 109 | m | DRB1*03, DQB1*0201, DQA1*0501 | Italy | 13,4 |  |  | + |
| 110 | f | DRB1*03, DQB1*0201, DQA1*0501 | Italy | 11,7 | Autoimmune thyroiditis |  | + |
| 111 | m | DRB1*03, DQB1*0201, DQA1*0501 | Italy | 10,0 |  |  | + |
| 112 | f | DRB1*03, DQB1*0201, DQA1*0501 DRB1*0417, DQB1*0302, DQA1*03 | Italy | 4,7 | Celiac disease |  | + |
| 113 | f | DRB1*03, DQB1*0201, DQA1*0501 DRB1*0401, DQB1*0302, DQA1*03 | Italy | 1,0 |  |  | + |
| 114 | m | DRB1*03, DQB1*0201, DQA1*0501 | Italy | 1,6 |  |  | + |
| 115 | f | DRB1*03, DQB1*0201, DQA1*0501 DRB1*0402, DQB1*0302, DQA1*03 | Albania | 12,1 |  |  | + |
| 116 | m | DRB1*03, DQB1*0201, DQA1*0501 | Italy | 14,2 |  |  | + |
| 117 | m | DRB1*0402, DQB1*0302, DQA1*03 DRB1*0405, DQB1*0302, DQA1*03 | Italy | 9,7 |  |  | + |
| 118 | f | DRB1*03, DQB1*0201, DQA1*0501 | Italy | 5,1 |  |  | + |
| 119 | m | DRB1*03, DQB1*0201, DQA1*0501 | China | 14,5 |  |  | - |
| 120 | f | DRB1*0402, DQB1*0302, DQA1*03/X | Italy | 8,7 |  |  | + |
| 121 | m | DRB1*03, DQB1*0201, DQA1*0501 | Morocco | 1,2 |  |  | + |
| 122 | f | DRB1*03, DQB1*0201, DQA1*0501 | Morocco | 8,2 |  |  | + |
| 123 | f | DRB1*03, DQB1*0201, DQA1*0501 | Italy | 8,9 |  |  | + |
| 124 | f | DRB1*01;07 DQB1*02;05 | Italy | 15,1 |  |  | + |
| 125 | m | DRB1*0403, DQB1*0302, DQA1*03 DRB1*03, DQB1*0201, DQA1*0501 | Italy | 11,5 | Celiac disease | T1DM familiarity (father) | + |
| 126 | m | DRB1*07;11 DQB1*02;03 | Italy | 7,5 | Autoimmune thyroiditis |  | + |
| 127 | f | DRB1*01;07 DQB1*02;05 | Italy | 13,7 |  |  | + |
| 128 | m | DRB1*01;07 DQB1*02;05 | Italy | 12,4 |  |  | + |
| 129 | m | DRB1*04;14 DQB1*03;05 | Italy | 16,1 |  |  | not available |
| 130 | m | DRB1*13;16 DQB1*05;06 | Italy | 16,0 |  |  | + |
| 131 | m | DRB1*07;13 DQB1*02;06 | Italy | 14,7 |  |  | + |
| 132 | f | DRB1*07;13 DQB1*02;06 | Italy | 5,4 |  | T1DM familiarity (father) | + |
| 133 | m | DRB1*01;07 DQB1*02;05 | Italy | 8,5 | Autoimmune thyroiditis |  | + |
| 134 | f | DRB1*07;13 DQB1*02;06 | Italy | 9,0 | Autoimmune thyroiditis |  | + |
| 135 | m | DRB1*11 DQB1*03;03 | Italy | 6,0 |  |  | - |
| 136 | f | DRB1*04;07 DQB1*02;04 | Not known | 11,8 |  |  | + |
| 137 | f | DRB1*07;08 DQB1*02;04 | Italy | 8,6 |  |  | + |
| 138 | f | DRB1*01;07 DQB1*02;05 | Italy | 15,6 |  |  | + |
| 139 | m | DRB1*04;16 DQB1*02;05 | Italy | 4,1 |  |  | + |
| 140 | m | DRB1*01;07 DQB1*03;05 | Italy | 13,2 | Autoimmune thyroiditis |  | + |
| 141 | f | DRB1*01;04 DQB1*02;05 | Italy | 5,7 |  | T1DM familiarity (mother) | + |
| 142 | m | DRB1*01;07 DQB1*03;05 | Italy | 11,2 |  |  | + |
| 143 | f | DRB1*11;12 DQB1*03 | Italy | 13,5 | Autoimmune thyroiditis |  | - |
| 144 | m | DRB1*04;07 DQB1*02 | Italy | 7,5 | Celiac disease |  | not available |
| 145 | m | DRB1*0403, DQB1*0302, DQA1*03 DRB1*03, DQB1*0201, DQA1*0501 | Italy | 17,2 |  |  | + |
| 146 | m | DRB1*01;04 DQB1*02;05 | Germany | 2,4 |  |  | + |
| 147 | f | DRB1*01;16 DQB1*05 | Italy | 11,7 |  |  | not available |
| 148 | f | DRB1*16 DQB1*05 | Italy | 6,1 | Autoimmune thyroiditis |  | + |
| 149 | m | DRB1*01;16 DQB1*05 | Italy | 12,7 |  |  | + |
| 150 | m | DRB1*04;13 DQB1*02;03 | Egypt | 5,8 | Autoimmune thyroiditis |  | + |
| 151 | f | DRB1*07;11 DQB1*02;03 | Italy | 6,8 | Celiac disease |  | + |
| 152 | m | DRB1*11;16 DQB1*05 | Italy | 8,4 |  |  | + |
| 153 | f | DRB1*07;11 DQB1*02;03 | Italy | 17,5 | Autoimmune thyroiditis |  | + |
| 154 | f | DRB1*09;13 DQB1*03;06 | Italy | 2,5 |  |  | + |
| 155 | f | DRB1*04;13 DQB1*02;06 | Italy | 4,3 |  |  | + |
| 156 | f | DRB1*01;16 DQB1*05 | Italy | 11,5 |  |  | not available |
| 157 | m | DRB1*04;07 DQB1*02;04 | Italy | 2,9 |  |  | + |
| 158 | m | DRB1*04;11 DQB1*03 | Italy | 13,7 |  |  | not available |
| 159 | f | DRB1*07;08 DQB1*03;04 | Albania | 9,6 | Psoriasis |  | - |
| 160 | f | DRB1*0403, DQB1*0302, DQA1*03 DRB1*03, DQB1*0201, DQA1*0501 | Italy | 9,4 |  |  | + |
| 161 | m | DRB1*07;11 DQB1*02;03 | Italy | 12,6 |  |  | + |
| 162 | f | DRB1*0405, DQB1*02, DQA1*03/X | Italy | 10,3 |  |  | - |
| 163 | m | DRB1*03, DQB1*0201, DQA1*0501 | Italy | 9,5 |  |  | + |
